# Species-specific viability analysis of *Pseudomonas aeruginosa*, *Burkholderia cepacia* and *Staphylococcus aureus* in mixed culture by flow cytometry

**DOI:** 10.1186/1471-2180-14-56

**Published:** 2014-03-07

**Authors:** Marc Rüger, Mandy Ackermann, Udo Reichl

**Affiliations:** 1 Chair of Bioprocess Engineering, Otto von Guericke University, Magdeburg, Germany; 2 Bioprocess Engineering, Max Planck Institute for Dynamics of Complex Technical Systems, Magdeburg, Germany

**Keywords:** Viability analysis, Mixed communities, Flow cytometry, T-RFLP, Cystic fibrosis, *Pseudomonas aeruginosa*, *Burkholderia cepacia*, *Staphylococcus aureus*, Interspecies effects

## Abstract

**Background:**

Bacterial species coexist commonly in mixed communities, for instance those occurring in microbial infections of humans. Interspecies effects contribute to alterations in composition of communities with respect to species and thus, to the course and severity of infection. Therefore, knowledge concerning growth and viability of single species in medically-relevant mixed communities is of high interest to resolve complexity of interspecies dynamics and to support development of treatment strategies. In this study, a flow cytometric method was established to assess the species-specific viability in defined three-species mixed cultures. The method enables the characterization of viability of *Pseudomonas aeruginosa*, *Burkholderia cepacia* and *Staphylococcus aureus*, which are relevant to lung infections of Cystic Fibrosis (CF) patients. The method combines fluorescence detection by antibody and lectin labeling with viability fluorescence staining using SYBR®Green I and propidium iodide. In addition, species-specific cell enumeration analysis using quantitative terminal restriction fragment length polymorphisms (qT-RFLP) was used to monitor the growth dynamics. Finally, to investigate the impact of substrate availability on growth and viability, concentrations of main substrates and metabolites released were determined.

**Results:**

For each species, the time course of growth and viability during mixed culture cultivations was obtained by using qT-RFLP analysis in combination with flow cytometry. Comparison between mixed and pure cultures revealed for every species differences in growth properties, e.g. enhanced growth of *P. aeruginosa* in mixed culture. Differences were also observed for *B. cepacia* and *S. aureus* in the time course of viability, e.g. an early and drastic reduction of viability of *S. aureus* in mixed culture. Overall, *P. aeruginosa* clearly dominated the mixed culture with regard to obtained cell concentrations.

**Conclusions:**

In combination with qT-RFLP analysis, the methods enabled monitoring of species-specific cell concentrations and viability during co-cultivation of theses strains. Experimental findings suggest that the predominance of *P. aeruginosa* over *B. cepacia* and *S. aureus* in mixed culture under the chosen cultivation conditions is promoted by more efficient substrate consumption of *P. aeruginosa*, and antagonistic interspecies effects induced by *P. aeruginosa*.

## Background

Microbial infections in humans are often characterized by a highly complex community of multiple bacterial species. Interspecies effects contribute to alterations in the composition of communities with respect to species and thus, to the course and severity of microbial infections. Moreover, changes in the composition of communities and interspecies effects may affect efficacy of antibiotics with severe consequences on therapeutic success. Consequently, determination of the dynamics of individual species in mixed communities is of high interest. Therefore, not only absolute and specific cell enumeration is required, but also information regarding viability of species. The composition of bacterial mixed communities can be determined efficiently by terminal restriction fragment length polymorphisms (T-RFLP) analysis. This has been widely demonstrated in numerous studies [1-6]. However, only few reports present absolute and species-specific cell numbers. For quantitative characterization of mixed communities by T-RFLP, Trotha et al. introduced an internal quantification standard [7], an 16S ribosomal RNA (rRNA) gene fragment from a known species with a defined cell number. Schmidt et al. adapted this quantitative T-RFLP (qT-RFLP) method for species-specific cell enumeration of a three-species mixed model community, comprising *P. aeruginosa*, *B. cepacia* and *S. aureus*, relevant to infections of lung of Cystic Fibrosis (CF) patients [8]. Furthermore, the authors characterized the growth of these species in a defined mixed culture in chemostat cultivations, and a mathematical chemostat model was established to identify interspecies effects [9]. Finally, Riedele et al. used the qT-RFLP protocol to study the efficacy of the antibiotic ceftazidime on the growth of these species in mixed culture [10]. While qT-RFLP method allows to discriminate between species and to quantify cell concentration, viability is not determined. The latter, however, is of crucial importance for assessing antibiotic efficacy in case cell damage does not result in cell lysis but in loss of cellular functions, e.g. membrane integrity. Only based on the combination of qT-RFLP and species-specific viability analysis the detailed description of growth dynamics and the thorough characterization of interspecies effects are feasible.

T-RFLP can also be used for selective detection of the viable cell fraction, when the method is modified as recently reported by Rogers et al., who stained cells with propidium monoazide (PMA) prior to cell disruption [11]. This dye, which intercalates with DNA, is generally excluded from viable cells with intact membranes [12]. By photo activation, PMA binding leads to irreversible modification of DNA, thus preventing DNA of dead cells with an permeabilized membrane from amplification by PCR [11]. While the combination of standard T-RFLP with PMA-T-RFLP allows for discrimination between viable and dead cells, it has the disadvantage that it does not allow for identification of cells with a slightly damaged membrane, which may occur during growth due to short-term perforation of the cell wall during cell division and cell wall synthesis [13]. Moreover, the PCR-based quantification of viable cells using PMA staining can be affected by the presence of a high number of dead cells in the sample [14]. Due to these drawbacks a flow cytometric method for characterization of species-specific viability had been established. Flow cytometry provides a powerful tool for viability analysis of bacteria by applying fluorescent probes, which target or indicate specific cell functions such as respiratory activity, enzyme activity, substrate uptake, efflux pump activity, membrane potential or membrane integrity [15]. Additionally, species discrimination can be achieved by species-specific fluorescence labeling of cells, e.g. by immunofluorescence using monoclonal antibodies. Flow cytometry has been widely applied for viability determination of bacteria, mainly by assessing membrane integrity of cells (see review [13]). Often, a nucleic acid double-staining (NADS) method is used using propidium iodide (PI) for identification of dead cells, and SYBR®Green I for total cell staining [16]. This approach has been demonstrated successfully for a wide range of different species [17-23]. PI is generally excluded from cells with intact membranes, whereas it enters cells with permeabilized membranes, staining them by binding DNA as well as RNA [24]. Concomitantly, SYBRGreen I penetrates all cells and intercalates selectively with double-stranded DNA [25]. Therefore, fluorescence detection enables discrimination between viable and dead cells exhibiting an intact or permeabilized membrane, respectively. In contrast to PMA-T-RFLP, detection of cells with a slightly damaged membrane can be obtained after optimization of staining conditions, as recently demonstrated for *S. aureus*[26]. For determination of species-specific viability by flow cytometry, fluorescence species-specific detection is a key prerequisite. Bacterial species can be detected specifically by fluorescence labeling with antibodies or lectins that bind specifically to epitopes or molecules on cell surface, and by labeling with nucleotide probes that hybridize specifically to complementary strands of 16S rRNA. Latter technique is not recommended for use in combination with viability staining, since labeling requires permeabilization of membranes. Even though fluorescence labeling using specific antibodies (immunofluorescence labeling) and lectins has been widely established in flow cytometry, the combination with viability staining has been used only very rarely to determine the species-specific viability in mixed cultures (see review [27]).

In this study, a flow cytometric method is presented for assessment of the species-specific viability in defined three-species bacterial mixed cultures comprising *P. aeruginosa*, *B. cepacia* and *S. aureus,* relevant to infections of the lung of CF patients. The approach combines species-specific fluorescence detection by lectin and antibody labeling with viability fluorescence staining using SYBR Green I and PI. Additionally, for growth characterization in mixed culture, species-specific cell enumeration analysis using qT-RFLP was applied. Finally, to study the effect of substrate availability on growth and viability, concentrations of main substrates and metabolites released into culture medium were quantified using an enzymatic assay and high performance liquid chromatography (HPLC) analysis.

## Methods

### Bacterial strains

*Pseudomonas aeruginosa* PAO1 was supplied by Kathrin Riedel (Department of Microbiology, Technical University Munich, Germany). *Burkholderia cepacia* DSM 7288 was supplied by the German Collection of Microorganisms and Cell Cultures (DSMZ, Braunschweig, Germany). *Staphylococcus aureus* ATCC 29213 was supplied by Brigitte König (Department of Medical Microbiology, Otto von Guericke University, Magdeburg, Germany).

### Culture medium

For cultivation of bacteria, Gibco® cell culture basal medium powder M199 (without NaCO_3_) (Life Technologies, Carlsbad, CA, USA) buffered with phosphate was used. Additionally, nitrilotriacetic acid (NTA) was added to prevent precipitation. Briefly, 800 mL of ultrapure water, 16 mL of 0.25 mM NTA solution (Sigma-Aldrich, Steinheim, Germany) in 0.6 M NaOH and 25 mL of sodium potassium phosphate buffer (1.5 M NaH_2_PO_4_/K_2_HPO_4_, pH 7.0, Carl Roth, Karlsruhe, Germany) were mixed with the amount of powder indicated by the supplier and filled up to 1 L with ultrapure water [8].

### Cultivation conditions

Bacteria were grown in 250 mL wide-neck Erlenmeyer flasks incubated in a humidified orbital shake incubator (Kuehner, Birsfelden, Switzerland) at 37°C, rotation speed 200 rpm, eccentric radius 1.25 cm, and relative humidity of 85%. Cultivations of mixed and pure cultures were conducted. Inocula were prepared as described previously by Riedele and Reichl [10] from pure culture of each species. Briefly, cells were grown overnight in 20 mL of culture medium, subsequently harvested, centrifuged at 3,522 × g for 10 min at 4°C (Heraeus® Multifuge 1S-R, Thermo Scientific, Waltham, WA, USA), and washed with PBS (8 g/L NaCl, 0.2 g/L KCl, 1.15 g/L NaH_2_PO_4_, 0.2 g/L K_2_HPO_4,_ pH 7.4, Carl Roth, Karlsruhe, Germany). Afterwards, suspensions were centrifuged again (3,522 × g, 10 min, 4°C), resuspended in 20 mL of fresh culture medium and cultivated for 1.5 h. These cells were inoculated in 50 mL fresh pre-warmed culture medium. For both mixed and pure cultures, the inoculation volume of each species was adjusted to a total starting cell concentration of 1 × 10^6^ cells/mL. Therefore, optical density at 650 nm (OD_650_) (photometer Ultraspec 3000, Amersham Biosciences, Otelfingen, Switzerland) was adjusted for each species based on linear correlation between OD_650_ and cell concentration determined by flow cytometric absolute counting. Consequently, for mixed culture, the inoculation volume of each species was three times lower than for pure culture (ratio between starting cell concentrations of species corresponds to 1:1:1). Cells were then cultivated over a period of 32 h.

For testing staining conditions, species were cultivated in pure cultures. Cells were grown overnight in 20 mL of culture medium, washed and regrown in 20 mL of fresh culture medium as described before. In contrast to inocula preparation, cells were cultivated over a period of 5 h. From these cultures samples were taken after 2 h and 5 h (exponential and stationary phase) for testing staining.

All cultivations were conducted in three biological replicates.

### Sample preparation before staining

Cells were harvested by centrifugation at 4,700 × g for 10 min at 4°C (Heraeus® Fresco, Thermo Scientific, Waltham, WA, USA) and subsequently resuspended in Ringer solution (39 mM NaCl, 1.4 mM KCl, 0.6 mM NaHCO_3_, 0.5 mM CaCl_2_•2H_2_O, pH 7.2, Carl Roth, Karlsruhe, Germany). For fluorescence staining, Ringer solution was supplemented with 0.05 mg/mL glutaraldehyde (GTA) (50% grade I, Sigma-Aldrich, Steinheim, Germany), 0.05% (m/v) BSA (Carl Roth, Karlsruhe, Germany), and 3 M KCl (Carl Roth, Karlsruhe, Germany) (pH 7.2). For analysis, samples were diluted with Ringer solution prior to staining if necessary to OD_650_ in a range of 0.01 to 0.04 to ensure total cell concentrations between 1 × 10^6^ and 1 × 10^7^ cells/mL.

To verify viability staining of dead cells, controls for membrane permeabilization (positive controls) were prepared for each species from pure cultures. Briefly, after resuspension in Ringer solution, samples were treated with 70% (v/v) isopropanol (Merck, Darmstadt, Germany) for 1 h at room temperature (RT). Subsequently, suspensions were centrifuged at 4,700 × g for 10 min at 4°C (see above) and washed. Then, cells were resuspended in Ringer solution and diluted to an OD_650_ in the range of 0.01 - 0.04.

### Labeling of WGA with Mix-n-Stain™ CF™405S

For Gram-staining of *S. aureus*, working solutions of 0.18 mg/mL CF™405S conjugated wheat germ agglutinin (WGA) were prepared. Briefly, 75 μg of WGA (Vector Laboratories, Burlingame, CA, USA), diluted in ultrapure water with 0.1 M NaHCO_3_ (Carl Roth, Karlsruhe, Germany), were labeled with Mix-n-Stain™ CF™405S antibody labeling kit (Biotium Inc., Hayward, CA, USA) according to the protocol recommended by the manufacturer.

### Antibodies for immunofluorescence detection of *B. cepacia*

For indirect immunofluorescence labeling of *B. cepacia*, primary monoclonal antibodies (1°Ab, mouse anti-Pseudomonas cepacia, IgG3, clone 1144/209, Biotrend Chemikalien GmbH, Köln, Germany) and R-phycoerythrin (R-PE) conjugated secondary monoclonal antibodies (2°Ab, goat anti-mouse, IgG, Life Technologies, Carlsbad, CA, USA) were applied. For every day of experiment, fresh working solutions of 10 μg/mL 1°Ab and 60 μg/mL 2°Ab were prepared in Ringer solution.

### SYBR Green I and PI for viability staining

For staining, working solutions of 1:100 SYBR®Green I (10,000 × concentrate in DMSO, Life Technologies, Carlsbad, CA, USA) and 1 mg/mL propidium iodide (Sigma-Aldrich, Steinheim, Germany) were prepared in ultrapure water.

### Four-color staining for viability assessment in mixed culture

Cells were fluorescently stained with established four-color staining protocol. Briefly, after preparation, cell suspensions were incubated with 20 μg/mL WGA-CF405S for 15 min in the dark at RT. Thereafter, suspensions were washed twice applying centrifugation at 4,700 × g for 10 min at 4°C (Heraeus® Fresco, Thermo Scientific, Waltham, WA, USA) and resuspension in Ringer solution. Subsequently, pelleted cells were resuspended in working solution of 1°Ab (10 μg/mL, final) and incubated for 1 h on ice. Afterwards, suspensions were washed as described above. Thereafter, cells were resuspended in working solution of R-PE conjugated 2°Ab (60 μg/mL, final) and incubated for 1 h on ice. After washing, cells were resuspended in Ringer solution and incubated simultaneously with SYBR Green I (dilution 5 × 10^3^) and PI (5 μg/mL) for 20 min in the dark at RT.

In addition, SYBR Green I/PI double-stained controls were processed for each species (same washing procedure as described above). For this, untreated as well as isopropanol-treated cells (positive controls) from both exponential (t = 4 h) and stationary growth phases (t = 12 h) were used.

### Flow cytometry

In this study, a CyFlow® space flow cytometer (Partec, Münster, Germany) equipped with a 16 mW 375 nm UV diode laser and a 20 mW 488 nm argon solid state laser was used for flow cytometric analysis. Forward (FSC) and side scatter (SSC) signals were collected at 488 nm. To reduce electronic background noise during analysis, SSC was defined as a discriminator. Threshold value was set to a lower limit of 300 (channel value) at a voltage of SSC of 260 mV. Fluorescence of WGA-CF405S was excited by the 375 nm laser and collected through a 455/50 nm band pass (BP) filter. R-PE immunofluorescence, SYBR Green I and PI fluorescence were excited by the 488 nm laser and collected through a 590/50 nm BP, 527/30 nm BP or a 630 nm long pass filter, respectively. Detected signals were amplified logarithmically (4 decades).

Degassed ultrapure water was applied as sheath fluid. Analysis was carried out with a sampling rate in the range of 100 to 1,000 particles/s. Samples were diluted accordingly with sheath fluid prior to analysis. To increase statistical significance, the total number of particles analyzed was set to 20,000 events for pure culture and to a minimum of 20,000 events for mixed culture samples, respectively. Data was collected with FloMax software (Version 2.70, Partec, Münster, Germany).

### Flow cytometric data analysis

Analysis, compensation and gating of data were performed using FlowJo software (Version 7.6.4, Tree Star Ashland, OR, USA). Due to spectral overlapping, fluorescence signals of SYBR Green I, R-PE and PI were compensated. Therefore, for every species, single-stained controls were processed in each experiment with the respective dye or fluorescence conjugate. PI single-stained controls were performed with isopropanol-treated cells. Compensation matrices were determined based on positive fluorescence signals of single-stained controls and subsequently applied for data analysis.

Flow cytometric data is presented in 2-D graphs either in pseudo color dot plots or in 5% quantile contour plots. Fluorescence intensities are given as relative fluorescence units (RFU).

For determination of species-specific viability, manually set gates were applied based on control samples. To discriminate cells from background, a gate for cell detection was applied. Only events with SYBR Green I positive fluorescence (intensities ≥ 1 RFU) were referred to as cells. Single species were detected based on WGA-CF405S fluorescence and R-PE immunofluorescence signals using a fixed gate for species discrimination. Subsequently, for each species, viability subpopulations were detected based on SYBR Green I and PI fluorescence signals. Applied viability gates were defined separately for each species for each exponential and stationary growth phase. Taking WGA-associated aggregation of *S. aureus* cells into account, for this strain, an additional gate for viability detection of single cells was used. This gate was set manually based on scatter signals (SSC vs FSC) of a SYBR Green I/PI double-stained sample from pure culture, which showed predominantly single cells.

### Species-specific cell concentrations by qT-RFLP

Growth of pure and mixed culture was determined by quantitation of species-specific cell concentrations using qT-RFLP analysis. Samples were treated and analyzed according to the description of Schmidt et al. [8]. Briefly, two samples were taken at each sampling point during cultivation and analyzed in parallel. 1 mL of sample was mixed with an internal quantification standard (IQS), an aliquot of *Campylobacter jejuni* with a fixed cell concentration. DNA of mixed sample was extracted by enzymatic cell lysis followed by DNA purification applying a commercially available kit (QIAamp DNA Blood Mini Kit, QIAGEN, Hilden, Germany). 16S rRNA genes were amplified by a gene-specific and fluorescently labeled primer using Polymerase Chain Reaction (PCR) method. Amplicons were then purified using an agarose gel and a commercially available extraction kit (QIAquick Gel Extraction Kit, QIAGEN, Hilden, Germany). Subsequently, amplicons were restricted with an HhaI endonuclease (R0139L, New England Biolabs Inc., Ipswich, MA, USA). Finally, species-specific rDNA fragments were separated by capillary gel electrophoresis and detected by laser-induced fluorescence (CGE-LIF) using a Genetic Analyzer ABI Prism 3100 Avant (Life Technologies, Carlsbad, CA, USA).

Species-specific cell concentration was determined by calculating the ratio of detected peak area for the given species to the peak area of the IQS [7,8]. Concentrations were declared as genome equivalents per mL (ge/mL) as described previously by Riedele and Reichl [10].

### Analysis of substrates and metabolites

Samples were harvested by centrifugation at 16,200 × g for 10 min at 4°C (Heraeus® Fresco, Thermo Scientific Waltham, WA, USA). Supernatants were withdrawn and filtrated using a syringe (Injekt 2 mL, B. Braun AG, Melsungen, Germany) with a 0.2 μm filter (Spartan 13/RC, Whatman GmbH, Dassel, Germany), and stored at 4°C until analysis.

Extracellular concentrations of glucose, glutamate and glutamine in the culture medium were determined according to a protocol of Riedele and Reichl [28] with a validated enzymatic quantification method using a Bioprofile 100 Plus (Nova Biomedical, Waltham, MA, USA). Quantitation limits and standard deviation (SD) of the method were as follows: glucose: 1.022 mM, SD 0.100 mM; glutamate: 0.254 mM, SD 0.025 mM; glutamine: 0.162 mM, SD 0.016 mM.

Extracellular concentrations of metabolites 2-keto-D-gluconic acid (2-KDG) and gluconic acid released in the culture medium, were quantified by HPLC (Agilent 1200 series, Agilent Technologies, Inc., Santa Clara, CA, USA) equipped with a Rezex-ROA organic acid column 300 × 7.6 mm (Phenomenex, Aschaffenburg, Germany). The applied method was in agreement with the method used by Riedele and Reichl [28] and comprised 0.075 M H_2_SO_4_ as eluent with a flow rate of 0.5 mL/min. From each sample 10 μL were injected and separation was performed at 45°C (column). Metabolites were detected by a diode array detector (DAD G1315B, Agilent Technologies, Inc., Santa Clara, CA, USA) at 210 nm. Quantitation limits and SD of the method were as follows: 2-KDG: 0.155 mM, SD 0.016 mM; gluconic acid: 0.267 mM, SD 0.026 mM.

### Statistical testing

To compare results obtained under different (mixed versus pure culture) or identical cultivation conditions, either unpaired or paired Student *t*-tests (p < 0.05) were performed, respectively.

## Results

### Characterization of viability using flow cytometry

**Figure 1:**
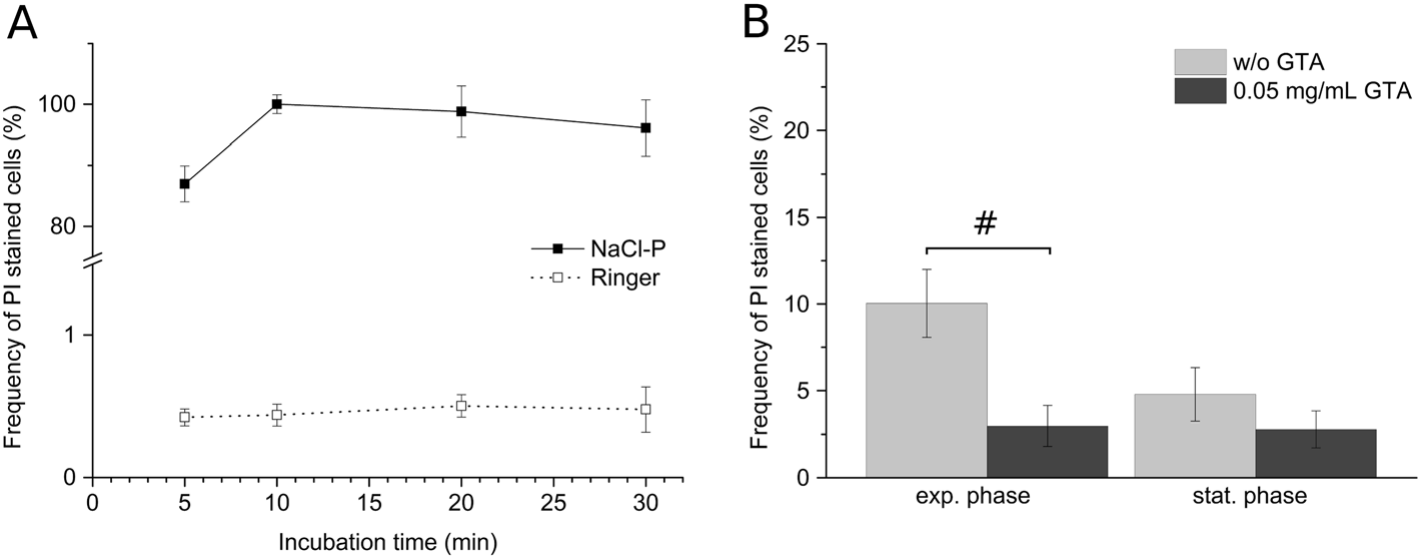
**Comparison of NaCl-P buffer and Ringer solution in viability determination of *P. aeruginosa *by flow cytometry. (A)** Relative frequencies of PI stained cells (5 μg/mL) for stationary grown *P. aeruginosa* from pure culture in NaCl-P buffer and Ringer solution at different dye incubation times. **(B)** Relative frequencies of PI stained cells in exponential and stationary growth phases from pure culture prepared in Ringer solution without GTA and with 0.05 mg/mL GTA. Relative frequency of PI stained cells was determined by ratio of relative frequency of PI stained events in untreated and isopropanol-treated samples. Error bars represent standard deviation of three biological replicates; # indicates a statistically significant difference (p < 0.05, paired Student *t*-test).

**Figure 2:**
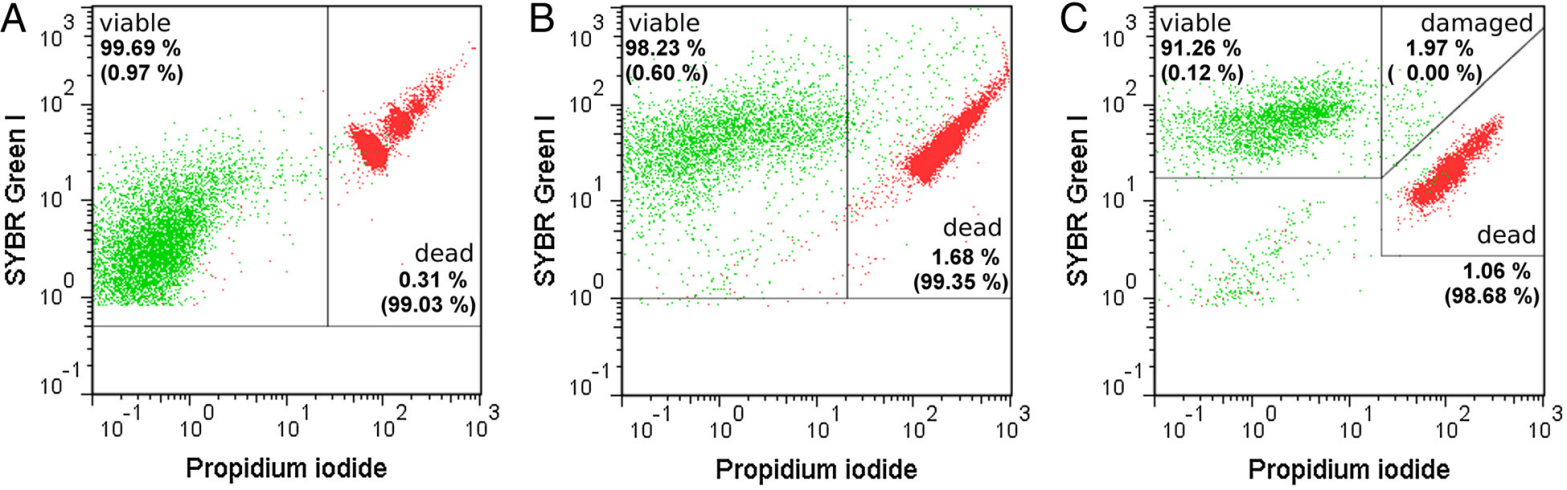
**Viability analysis by flow cytometry.** Viability was determined by membrane integrity analysis using SYBR Green I (dilution of 5 × 10^3^) and PI (5 μg/mL). Representative pseudo color dot plots with defined gates are shown for **(A)***P. aeruginosa*, **(B)***B. cepacia* and **(C)***S. aureus* in stationary growth phase (t = 12 h) from pure culture. Gates were set manually for each species based on SYBR Green I and PI fluorescence signals of isopropanol-treated (red) and untreated cells (green). For each gate region, relative frequencies of total cells are presented. Only events with positive SYBR Green I fluorescence were considered as cells (see Figure 7).

**Figure 3:**
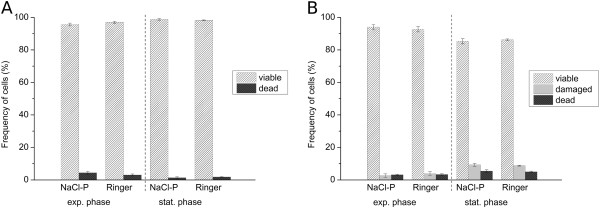
**Comparison of NaCl-P buffer and Ringer solution for viability analysis of *B. cepacia *and *S. aureus *by flow cytometry.** Viability was determined by membrane integrity analysis using SYBR Green I (dilution of 5 x 10^3^) and PI (5 μg/mL). Relative frequencies of **(A)** viable and dead cells of *B. cepacia* and **(B)** of viable, damaged and dead cells of *S. aureus* in pure culture from both exponential and stationary growth phase. Relative frequencies were determined based on all SYBR Green I and PI fluorescence positive events. Error bars represent standard deviation of three biological replicates.

Viability was determined by membrane integrity analysis using flow cytometry. Therefore, nucleic acid double-staining (NADS) was applied, comprising PI for identification of dead cells and SYBR Green I for total cell staining. Staining conditions (5 μg/mL PI; 5 × 10^3^ SYBR Green I dilution; NaCl phosphate buffer containing 0.05 mg/mL GTA) were chosen according to an optimized method for viability analysis of *S. aureus* and *B. cepacia* in mixed culture published recently [26]. However, since PI staining of *P. aeruginosa* in NaCl phosphate buffer (NaCl-P) led to a total loss of membrane integrity (Figure 1(A)), this buffer was replaced by Ringer solution containing GTA. Remarkably, the addition of GTA did not impair membrane integrity of *P. aeruginosa* (Figure 1(B)). The modified double-staining protocol enabled reproducibly the detection of characteristic viability subpopulations of each species. Dead cells with permeabilized membranes and viable cells with intact membranes were detected based on green and red fluorescence (Figure 2). Viable cells displayed SYBR Green I fluorescence and no PI fluorescence, whereas dead cells showed intense PI fluorescence as well as SYBR Green I fluorescence. Remarkably, fluorescence intensities varied between species after staining, particularly with respect to SYBR Green I. The existence of dead cells was verified by staining of cells treated with 70% (v/v) isopropanol (positive controls), which resulted in identical fluorescence signals compared to the subpopulation of dead cells in untreated samples. For *S. aureus*, an additional subpopulation was detected, which was referred to as “cells with slightly damaged membranes” (damaged) as described previously [26]. In comparison to the staining method established previously in NaCl phosphate buffer [26], similar values were determined for the frequency of viable and dead cells of *B. cepacia* (Figure 3(A)) as well as viable, damaged and dead cells of *S. aureus* (Figure 3(B)) in pure culture using Ringer solution.

### Species-specific viability analysis in mixed culture

**Figure 4:**
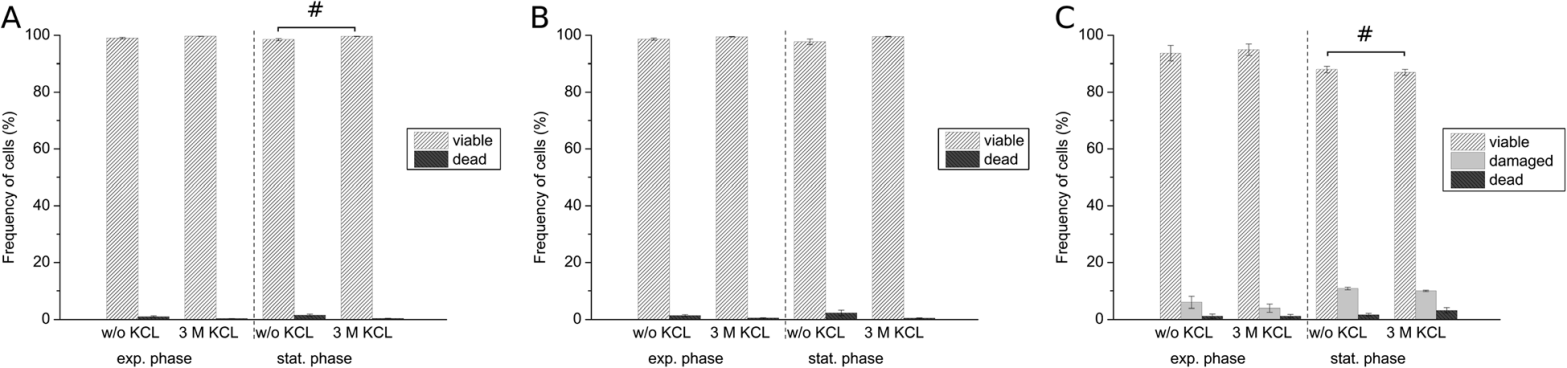
**Impact of KCL on flow cytometric viability determination.** Comparison of Ringer solution without and with 3 M KCl. Viability was determined by membrane integrity analysis using SYBR Green I (dilution of 5 × 10^3^) and PI (5 μg/mL). Relative frequencies of **(A)** viable and dead cells of *P. aeruginosa,***(B)** of viable and dead cells of *B. cepacia* and **(C)** of viable, damaged and dead cells of *S. aureus* in exponential and stationary growth phases in pure culture. Relative frequencies were determined based on all SYBR Green I and PI fluorescence positive events. Error bars represent standard deviation of three biological replicates; # indicates a statistically significant difference (p < 0.05, paired Student *t*-test).

**Figure 5:**
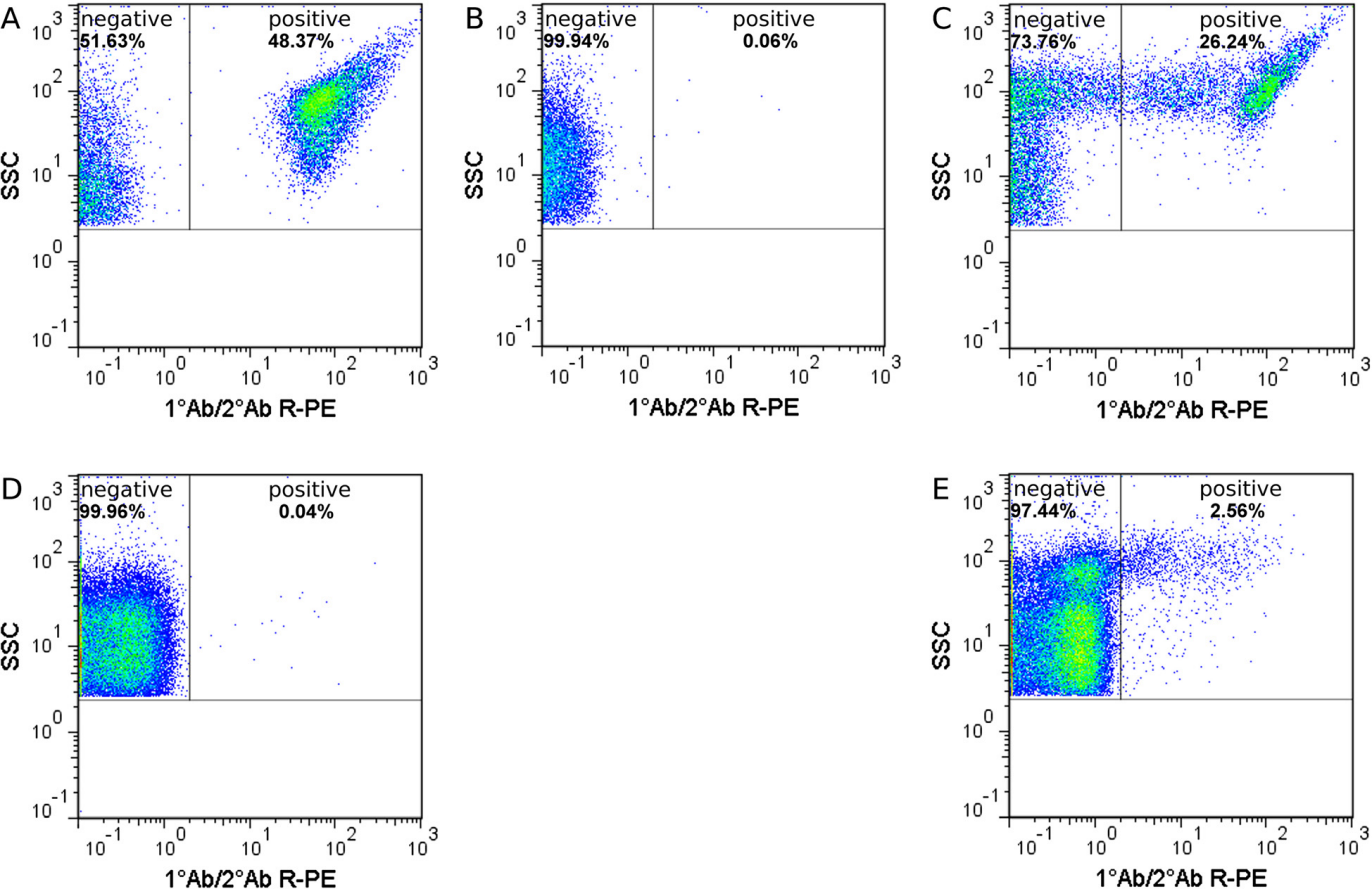
**Immunofluorescence testing by flow cytometry.** 5 μg/mL 1°Ab and 20 μg/mL R-PE conjugated 2°Ab were used representative pseudo color dot plots for **(A)***B. cepacia*, **(B)***P. aerugino*sa and **(C)***S. aureus* in exponential growth phase (t = 2 h) from pure cultures. Additionally for species with positive immunofluorescence signal using 1°Ab and 2°Ab, binding of 2°Ab alone was tested: **(D)***B. cepacia* and **(E)***S. aureus*. Gates were set manually based on R-PE fluorescence signal of unstained and stained cells. For each gate, relative frequencies of total events are presented.

**Figure 6:**
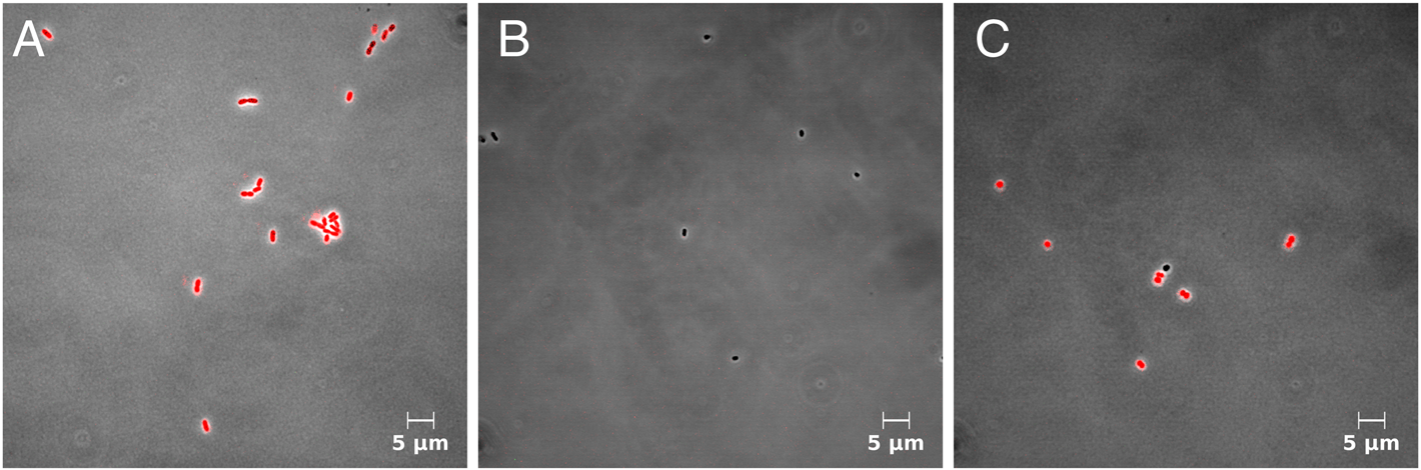
**Immunofluorescence testing by fluorescence microscopy.** 5 μg/mL 1°Ab and 20 μg/mL R-PE conjugated 2°Ab were used representative microscopic images for **(A)***B. cepacia*, **(B)***P. aerugino*sa and **(C)***S. aureus* in exponential growth phase (t = 2 h) from pure cultures. Laser Scanning Microscopy was employed (LSM 510, Carl Zeiss AG, Oberkochen, Germany). R-PE fluorescence was excited by 1 mW 543 nm Helium-Neon Laser and collected through a 585 nm long pass filter. Images are magnified 1000 x.

**Figure 7:**
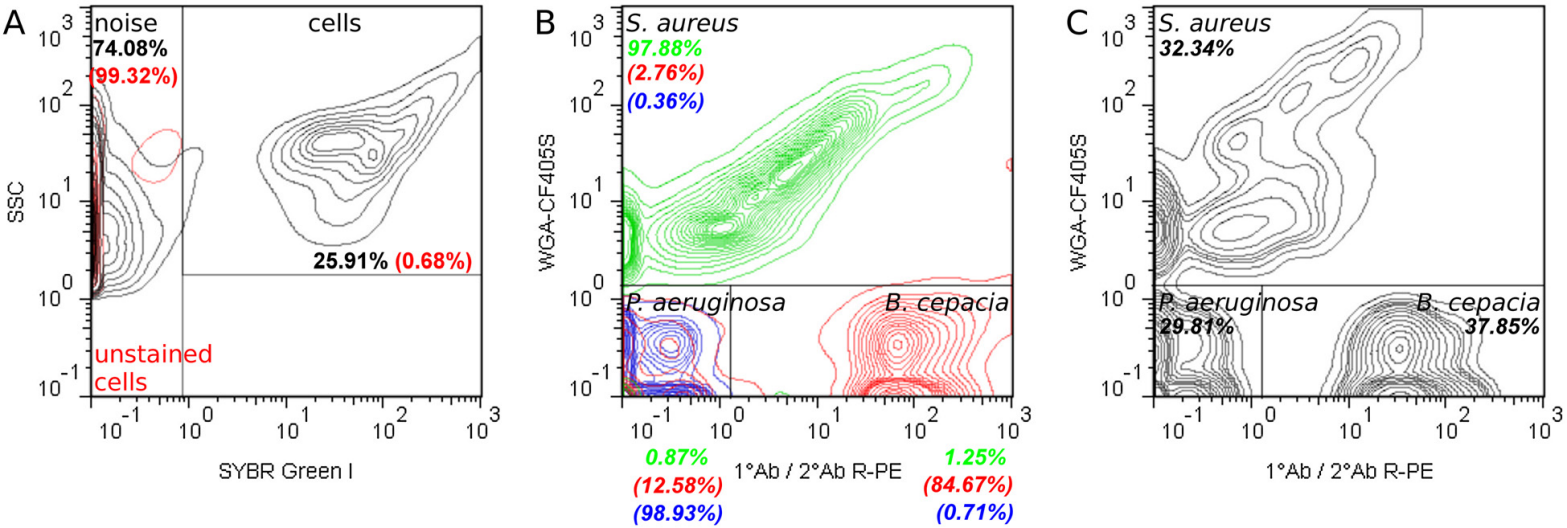
**Species discrimination in flow cytometric viability analysis.** Four-color staining was applied. Samples were incubated with 20 μg/mL WGA-CF405S, 10 μg/mL 1°Ab and 60 μg/mL R-PE conjugated 2°Ab, SYBR Green I (dilution of 5 × 10^3^) and 5 μg/mL PI. **(A)** For data analysis, only events with SYBR Green I positive fluorescence (cells) were considered. The cell gate was set manually based on the signal of four-color-stained (black) and unstained cells (red) in exponential growth phase (t = 4 h) from pure culture. **(B, C)** Gating for species discrimination in data acquired from mixed culture samples. Gate was set manually based on WGA-CF405S fluorescence and R-PE immunofluorescence signals of four-color-stained single species from pure culture. **(B)** Overlaid cytometric plot of *P. aeruginosa* (blue), *B. cepacia* (red) and *S. aureus* (green) in exponential growth phase (t = 4 h) from pure culture. **(C)** Gate applied for species discrimination in mixed culture sample at time point of inoculation. For each gate region, either relative frequencies of total events or cells (in italics) are shown. Data is presented in 5% quantile contour plots.

For determination of species-specific viability in defined mixed cultures, comprising three bacterial species relevant to lung infections of CF patients by flow cytometry, a four-color staining method was established. Therefore, a previously presented method for binary mixed cultures [26], comprising Gram-specific fluorescence labeling of *S. aureus* using CF405S-conjugated WGA in combination with NADS, was extended to enable indirect immunofluorescence labeling of *B. cepacia*. Two monoclonal antibodies were used, a primary antibody mouse anti-Pseudomonas cepacia (1°Ab) and a secondary antibody goat anti-mouse conjugated with R-PE (2°Ab). In contrast to the method published previously, Ringer solution was applied instead of NaCl-P buffer. For efficient discrimination of WGA CF405S labeled *S. aureus* from unlabeled *P. aeruginosa* and *B. cepacia*, 3 M KCL had to be added to the Ringer solution, which resulted in an increased fluorescence signal of WGA labeling as described previously in the study of Holm et al. [29]. That the addition of KCl did not impair membrane integrity of single species during analysis was tested beforehand by SYBR Green I and PI double-staining experiments using pure cultures. In particular, relative frequencies of viable and dead cells of *P. aeruginosa* and *B. cepacia*, and viable, damaged, and dead cells of *S. aureus* were generally similar in comparison to frequencies determined for references without addition of KCl (Figure 4). The reliability of immunofluorescence labeling for specific detection of *B. cepacia* was tested beforehand by single-staining experiments of pure culture samples using flow cytometry and fluorescence microscopy. For testing, a combination of 5 μg/mL 1°Ab and 20 μg/mL 2°Ab as well as use of 2°Ab alone were applied. Immunofluorescence testing revealed for 1°Ab efficient binding to *B. cepacia*, no binding to *P. aeruginosa* and unspecific binding to *S. aureus*. Moreover, 2°Ab did neither bind to *B. cepacia* nor to *P. aeruginosa*, but bound unspecifically to *S. aureus* (Figures 5 and 6). Thus, indirect immunolabeling of *B. cepacia* using both antibodies resulted in intense R-PE fluorescence staining of *B. cepacia*. However, in mixed culture, fluorescence signals of immunolabeled *S. aureus* interfered with immunofluorescence signals of *B. cepacia*. Despite of this, the tested immunolabeling procedure was demonstrated to be highly efficient for species detection in mixed culture; in particular, when combined with WGA-CF405S staining of *S. aureus* and total cell staining, with SYBR Green I and PI. For data analysis, only events with SYBR Green I positive fluorescence (cells) were considered (Figure 7(A)). Due to efficient Gram-specific staining of *S. aureus,* neither *P. aeruginosa* nor *B. cepacia* were stained with WGA-CF405S by applying four-color staining (Figure 7(B)). Therefore, all three species could be detected specifically in mixed culture by four-color staining by adequate discrimination according to WGA-CF405S fluorescence and R-PE immunofluorescence signals (Figure 7(C)). For reproducible and efficient species discrimination for all mixed culture samples, a fixed gate was applied, which was defined manually according to signals obtained in pure culture experiments (Figure 7(B)). As a result, the gating of four-color stained cells from pure culture enabled to obtain cell recoveries above 98% for *P. aeruginosa*, above 94% for *S. aureus* and above 93% for *B. cepacia* throughout the cultivation period covered in the performed cultivations (t = 32 h). Exceptions were found for *B. cepacia* during exponential growth phase (t = 4 h). Here, the cell recovery was only about 78% (mean value from three biological replicates). Overall, the species discrimination based on gating allowed then for efficient determination of species-specific viability in the three-species mixed culture according to SYBR Green I and PI fluorescence signals. For each species, relative frequencies of viability subpopulations were determined by respective species-specific gating (Figure 2).

### Assessment of growth and viability in pure and mixed culture cultivations

**Figure 8:**
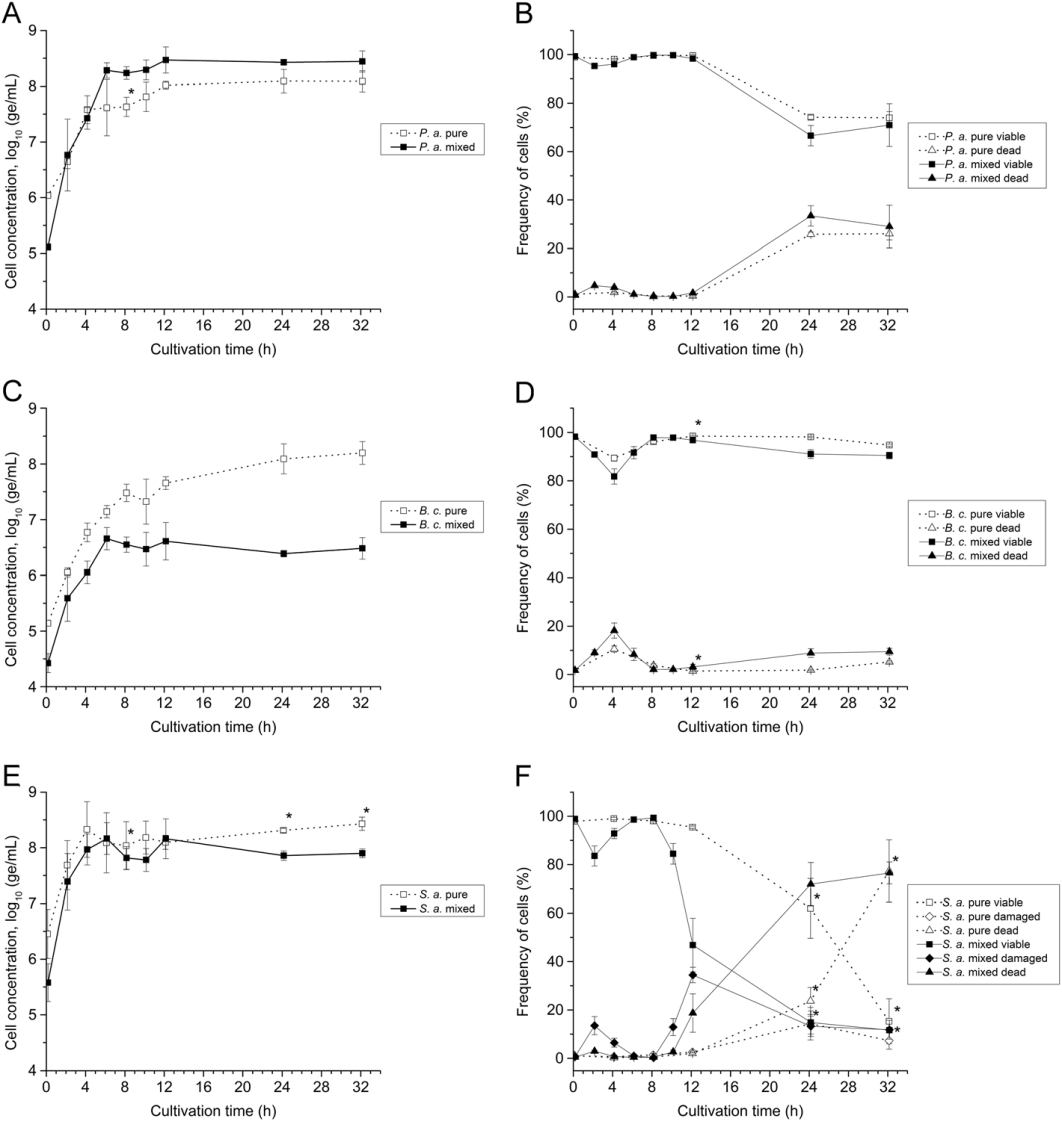
**Growth and viability in pure and mixed cultures.** Species-specific cell concentrations and viability were determined over a cultivation period of 32 h by qT-RFLP and flow cytometry, respectively. Open symbols represent data from pure cultures and filled symbols from mixed culture. Left: Dynamics of log_10_-transformed species-specific cell concentrations of **(A)***P. aeruginosa*, **(C)***B. cepacia*, and **(E)***S. aureus*. Right: Dynamics of relative frequencies of **(B)** viable and dead cells of *P. aeruginosa*, **(D)** viable and dead cells of *B. cepacia* and **(F)** viable, damaged and dead cells of *S. aureus*. Relative frequencies of viability subpopulations were determined based on all SYBR Green I and PI fluorescence positive events in the respective species gate. Subpopulations were defined as gated in plots shown in Figure 2. Error bars represent standard deviation of two (*) or three biological replicates.

Viability of *P. aeruginosa*, *B. cepacia* and *S. aureus* was assessed by membrane integrity analysis during growth in pure and mixed culture cultivations, using the established four-color staining method and flow cytometry. Additionally, to characterize growth of single species, species-specific cell concentrations were determined by qT-RFLP. Comparison between dynamics of cell concentrations in mixed and pure cultures revealed differences for every species in growth properties (Figure 8(A, C, E)). For *P. aeruginosa,* growth was improved in mixed culture. Cells grew faster with a significant higher maximum specific growth rate (p < 0.05, unpaired Student *t*-test; Table 1). With 6 h the exponential growth phase took also longer than in pure culture. Consequently, the maximum cell concentration (log_10_ 8.47 ± 0.23 ge/mL) was higher than in pure culture (Figure 8(A)). For *B. cepacia*, growth dynamics were similar for the first 6 h of cultivation in pure and mixed culture (Figure 8(C)). Also, the maximum specific growth rates were comparable (Table 1). However, after 6 h time courses differed significantly from each other. In mixed culture, growth remained static until 12 h before cell concentration decreased slightly towards the end of cultivation. In contrast, in pure culture, cell concentration increased until the end of experiment. The maximum cell concentration in pure culture (log_10_ 8.20 ± 0.20 ge/mL) was almost two-log levels higher than in mixed culture (Figure 8(C)). For *S. aureus*, time course of cell concentrations were comparable for the first 12 h of cultivation in pure culture and mixed culture (Figure 8(E)). Maximum specific growth rates (Table 1) as well as maximum cell concentrations were also similar. However, after 12 h time courses differed from each other. In mixed culture cell concentrations decreased slightly until the end of cultivation, whereas concentrations in pure culture remained relatively constant (Figure 8(E)).

**Table 1:** **Maximum specific growth rates μ**_**max **_**in pure and mixed culture**

**Species**	**Pure culture**		**Mixed culture**	
	**μ**_**max **_**(h**^**−1**^**)**	**Δt (h)**	**μ**_**max **_**(h**^**−1**^**)**	**Δt (h)**
*P. a.*	0.88 ± 0.11	0 - 4	1.22 ± 0.05	0 - 6
*B. c.*	0.77 ± 0.02	0 - 6	0.86 ± 0.09	0 - 6
*S. a.*	1.08 ± 0.37	0 - 4	1.29 ± 0.31	0 - 4

Parameters were determined for *P. aeruginosa*, *B. cepacia* and *S. aureus* by linear regression from log_e_ cell concentrations during exponential growth phase; mean values ± standard deviation from three biological replicates.

For *P. aeruginosa,* the viability in pure and mixed culture was initially high at about 99%. Then it remained relatively constant before decreasing after 12 h to achieve about 73% towards the end of cultivation (Figure 8(B)). For *B. cepacia*, viability in pure and mixed culture was as high as for *P. aeruginosa* in the beginning. However, viability was reduced during exponential growth phase after 4 h to about 89% in pure or 80% in mixed culture, respectively. Subsequently, viability increased in both cultures to a level of about 98% to remain constant from 8 h to 12 h (Figure 8(D)). Afterwards, viability decreased in both cultures slightly towards the end of cultivation. For *S. aureus*, the viability in pure and mixed culture was initially as high as determined for the Gram-negative bacteria. Viability differed slightly between pure and mixed culture during exponential growth phase (0 h to 4 h). However, at 8 h, viabilities achieved again similar levels of about 98%. Afterwards, the time course of viability of *S. aureus* differed significantly between pure and mixed cultures (Figure 8(F)). Although viability decreased in both cultures towards the end of cultivation, in mixed culture, it decreased earlier than in pure culture and initially at a faster rate. Briefly, viability in mixed culture dropped instantly from 8 h to 12 h to about 47%, whereas viability in pure culture remained high at about 97%. Afterwards, viability decreased continuously in both cultures to levels of about 13%. The frequency of dead cells of *S. aureus* in pure and mixed culture correlated well with the time course of viable cells over a cultivation period of 24 h or 12 h, respectively. Remarkably, for *S. aureus* in mixed culture, loss of viability resulted first in an increase of damaged cells and subsequent in an increase of dead cells, whereas in pure culture, damaged cells increased with dead cells. However, in both cultures, damaged cells decreased towards the end of cultivation, whereas dead cells continued increasing.

**Figure 9:**
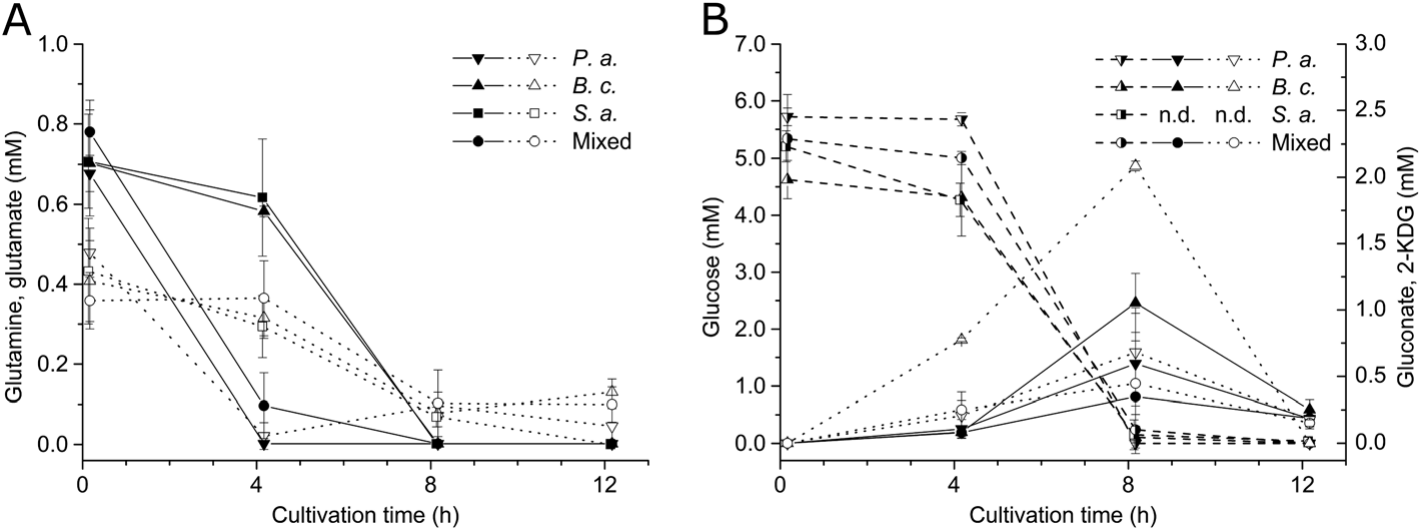
**Extracellular substrate and metabolite concentrations in pure and mixed cultures.** Glutamine, glutamate and glucose were quantified by an enzymatic assay. Gluconate and 2-KDG were quantified by HPLC. Time courses of **(A)** glutamine (filled symbols) and glutamate (open symbols) and **(B)** glucose (half-filled symbols), 2-KDG (filled symbols) and gluconate (open symbols) are presented over a cultivation period of 12 h. For *S. aureus* neither gluconate nor 2-KDG were detected (n.d., below detection limit). Error bars represent standard deviation of three biological replicates.

To investigate the impact of substrate availability on growth and viability of single species, concentrations of main substrates and metabolites released in the culture medium were quantified in pure and mixed cultures during the first 12 h of cultivation. Glucose, glutamine and glutamate were consumed completely by all species within 8 h in pure and mixed culture (Figure 9). Interestingly, glutamine and glutamate were consumed faster by *P. aeruginosa* than by *B. cepacia* and *S. aureus* in pure culture*.* In mixed culture, glutamine was consumed as fast as observed in pure culture of *P. aeruginosa*. Gluconate and 2-KDG were produced in pure cultures of *P. aeruginosa* and *B. cepacia* as well as in mixed cultures during the first 8 h of cultivation (Figure 9(B)). Remarkably, these metabolites were consumed by *P. aeruginosa* and *B. cepacia* in pure culture. Moreover, gluconate and 2-KDG were also consumed in mixed cultures.

## Discussion

### Species-specific determination of viability by flow cytometry

In this study, flow cytometry has been shown to be a highly capable technique for efficient determination of species-specific viability of *P. aeruginosa*, *B. cepacia* and *S. aureus* in defined mixed cultures relevant to lung infections of CF patients. Even though, a combination of the applied qT-RFLP method with PMA pre-treatment may enable detection of viable cells, in this study, the use of flow cytometry was preferred for viability analysis due to two practical limitations of PMA-T-RFLP: First, cells with slightly damaged membranes, which may occur during growth due to short-term perforation of the cell wall during cell division and cell wall synthesis [13], cannot be discriminated by PMA-T-RFLP from viable cells or dead cells, respectively. Second, quantification of viable cells by T-RFLP using PMA pre-treatment can be affected by the presence of a high number of dead cells in the sample (see review [14]), in particular, with a concentration of dead cells exceeding 10^4^ cells/mL [30-33].

Viability was determined by flow cytometry assessing the membrane integrity of cells using an adapted NADS protocol (see Methods). PI was applied for identification of dead cells in combination with SYBR Green I for total cell staining. For each species, the protocol enabled detection of dead and viable cells with permeabilized or intact membranes, respectively. It is assumed that the loss of membrane integrity is associated with missing reproductive growth and missing metabolic activity and eventually leads to cell death [34]. Thus, cells labeled as viable can be metabolically active or inactive as well as culturable or non-culturable cells. PI fluorescence signal intensities of dead cells increased most likely by quenching from SYBR Green I to PI when cells are double-stained [35]. Fluorescence intensities of viable and dead cells varied between species, which can be largely attributed to differences in cell wall structure [13], total DNA content and base composition [36], as well as in efflux pump activity [26]. Weak SYBR Green I fluorescence intensities, especially for viable cells of *P. aeruginosa*, suggest an out-pumping of dye from cells by active efflux pumps. In contrast, dead cells of *P. aeruginosa* exhibited intense SYBR Green I fluorescence, which is most likely due to missing efflux activity when membranes are permeabilized. For *S. aureus*, damaged cells were additionally detected, which were referred to as “cells with a slightly damaged membrane” by taking results of other authors [16,23] into account. These cells are probably stained with a low amount of PI resulting in incomplete quenching from SYBR Green I to PI [35].

For assessment of species-specific viability in mixed cultures, Gram-specific staining of *S. aureus* and indirect immunolabeling of *B. cepacia* using fluorescently labeled WGA and monoclonal antibodies, respectively, were employed successfully in combination with the adapted NADS protocol. However, the establishment of the method was very time consuming, since every single staining condition had to be tested separately for each bacterial species. Moreover, such an approach can only be applied for bacteria cultivable in pure culture, since labels and viability dyes need to be tested a priori for each species. Thus, the use of such technique is restricted to defined mixed cultures. The established four-color staining method allowed for efficient specific detection of *P. aeruginosa*, *B. cepacia* and *S. aureus* in mixed culture samples and subsequent determination of viability. WGA was shown to bind exclusively to *S. aureus*, most likely to the outer peptidoglycan layer of the cell wall, since it is known to bind specifically to N-acetylglucosamine and N-acetylneuroaminic residues [37]. The fact that WGA does not bind to Gram-negative bacteria in mixed culture is most probably attributed to the presence of an outer membrane [38]. WGA staining of *S. aureus* was as efficient and reproducible as presented in a previously published report for specific detection of *S. aureus* in binary mixed cultures [26]. Moreover, the binding capacity of WGA remained stable over the time period covered in the conducted cultivations, as demonstrated in pure culture. Indirect immunolabeling allowed for efficient specific detection of *B. cepacia* in mixed culture samples, when four-color staining method was applied. Cross reaction of both monoclonal antibodies to *S. aureus* was also observed, and is most probably attributed to the presence of protein A in the cell wall of this strain. Protein A is known to react with antibodies [39,40], particularly with high affinity to immunoglobulin G [41]. Specific detection of *B. cepacia* by immunolabeling was very efficient and reproducible, but reduced recoveries were observed during the exponential growth phase. This may be explained by low accessibility of surface-exposed epitopes at proliferation. This is in agreement with conclusions made from insufficient immunofluorescence labeling of bacteria in a report of Hughes et al., who suggested a cell cycle-dependent exposure of the targeted outer membrane protein of the species of interest [42]. Overall, the cell recoveries of specifically detected species were very high for the obtained samples throughout the cultivations, as demonstrated for pure cultures. However, they were less than 100%. Accordingly, with respect to species-specific labels, false negative counts of *B. cepacia* and *S. aureus* cells cannot be excluded completely. Consequently, this may have a slight impact on the detected relative frequencies of viable and dead *P. aeruginosa.* However, for the purpose of this study describing the viability dynamics in mixed culture, the specificity of this protocol was sufficiently high. For other purposes a third species-specific label may be advisable.

### Mixed culture effects on viability and growth

To investigate mixed culture effects on viability and growth of the three single species, mixed culture as well as pure culture cultivations were performed. Species-specific viability was assessed using the established flow cytometric method. Growth of single species was assessed by quantification of species-specific cell concentrations using qT-RFLP analysis. To investigate a possible impact of cell concentrations at time point of inoculation onto dynamics in the mixed culture, cultivations were carried out with one-log-reduced starting cell concentrations in comparison to cultures conducted in the study of Riedele and Reichl [10]. However, the ratio between species in mixed culture was maintained.

For all species, analysis of cell concentrations by qT-RFLP revealed clear differences in growth dynamics between mixed and pure culture. *P. aeruginosa* showed improved growth in mixed culture. According to conclusions drawn by Riedele and Reichl from their experiments this extended exponential growth phase is most likely due to the reduced starting cell concentrations in mixed culture compared to pure culture (see Methods), which enabled an increasing number of cell divisions while the substrate is consumed until depletion [10]. This is supported by the finding that levels of glutamine and glucose dropped below the detection limit after 4 h or 8 h, respectively, following comparable time profiles in mixed and pure culture. Furthermore, in both cultures, level of gluconate dropped below the detection limit after 4 h or 8 h, respectively. However, the higher specific growth rate in mixed culture compared to pure cultures may be caused by an unknown interspecies effect and remains to be identified. In case of *B. cepacia,* growth stagnated in mixed culture after 6 h of cultivation, whereas growth in pure culture was observed until the end of cultivation. These differences may be explained partially by competition for glutamine with *P. aeruginosa* during growth. This is supported by the finding that glutamine was consumed in mixed culture as fast as in pure culture of *P. aeruginosa* and thus, faster than in pure culture of *B. cepacia*. Furthermore, growth stagnation of *B. cepacia* in mixed culture may be caused by antagonistic interaction through action of inhibitory active substances against *B. cepacia*, e.g. pyocins, produced by *P. aeruginosa.* The latter could further explain the decrease of cell concentrations of *B. cepacia* in mixed culture towards the end of cultivation. In fact, the PAO1 strain is known to produce pyocins [43,44], and its inhibitory activity against *B. cepacia* was demonstrated in a study of Bakkal et al. [45], in which different clinical strains of *B. cepacia* and *P. aeruginosa* isolated from CF patients were tested in mixed culture. Although Riedele and Reichl [10] suggested in their study no significant alterations for cell doublings of *B. cepacia* between growth in pure and mixed culture, the cell concentrations clearly reveal the same trend towards the end of cultivation as observed in this study. However, the mode of action of inhibitory active substances against *B. cepacia* produced by *P. aeruginosa* remains to be elucidated. Despite of this, obtained results indicate no antagonistic interactions from *S. aureus* to *P. aeruginosa* or to *B. cepacia*. The latter is in agreement with findings of a previously published study, in which these strains were characterized in binary mixed cultures [26].

For *S. aureus*, mixed culture cultivations did not affect growth of single species. Interestingly, other authors reported inhibition of growth in similar mixed cultures [10,46], possibly caused by interspecies competition for glucose and glutamine [10] as well as through action of antistaphylococcal pyocyanine or pseudomonas quinoline signals (PQS) produced by *P. aeruginosa*[46-48]. Although the ratio between species used to initiate mixed culture experiments was similar to the study of Riedele et al. [10], in this study, growth characteristics of *S. aureus* were comparable between mixed and pure cultures. This is probably attributed to more favorable growth conditions for the Gram-positive bacteria in mixed culture at reduced starting cell concentrations of each species, e.g. due to the higher ratio of initial substrate-to-biomass [49]. However, *S. aureus* seems to be outcompeted during stationary growth phase, as cell concentrations decreased in mixed culture towards the end of cultivation. This may be explained by action of inhibitory and lytic active substances produced by the competing species. Especially, *P. aeruginosa* is known to produce antistaphylococcal virulence factors, either bactericidal active, e.g. phenazine pyocyanine [50,51], or bacteriolytic active, e.g. LasA protease [52]. This hypothesis is further supported by results obtained in a proteomic study published recently by Kluge et al., where induction of phenazine synthesis of *P. aeruginosa* was suggested due to increased expression levels of PhzS, PhzD and PhzG2 in a similar mixed culture [46]. Additionally, the authors found for *S. aureus* an increased expression level of an alkyl hydroperoxide reductase, which typically evokes resistance against oxidative stress. Despite of antistaphylococcal action of *P. aeruginosa* in mixed culture, it is likely, that virulence factors of *B. cepacia* also contribute to lysis of *S. aureus*, e.g. peptidoglycan hydrolases [53]. This interaction, however, still needs to be demonstrated. Cell concentrations of *B. cepacia* in pure and mixed culture determined by qT-RFLP might, however, be underestimated, in particular at time point of inoculation. Although cell concentrations were adjusted (see Methods), the determined values were up to one-log unit below concentrations measured for *P. aeruginosa* and *S. aureus*. In contrast, flow cytometry suggested for *B. cepacia*a cell concentration in mixed culture at time point of inoculation to as high as adjusted (see Methods). Detected relative frequencies revealed a ratio between starting cell concentrations of species of about 1:1:1 (Figure 7(C)).

In general, viability of species shifted over the cultivation period. Experimental data indicates that the loss of viability in pure culture during stationary phase, particularly for *P. aeruginosa* and *S. aureus*, is a consequence of the depletion of the main substrates glucose, glutamine and glutamate within the first 12 h of cultivation. Moreover, gluconate and 2-KDG were also depleted within this time period, which are intermediates produced via extracellular glucose oxidation by *P. aeruginosa* and *B. cepacia*[54,55]. With depletion of nutrients the metabolic activity decreases and active transports are impaired, which eventually leads to permeabilization of cytoplasmic membranes [34]. In contrast, viability of *B. cepacia* in pure culture remained relatively constant throughout the entire cultivation period, although the main substrates and metabolites were also depleted within the first 12 h of cultivation. Interestingly, in this phase, growth of *B. cepacia* was still detected by increasing cell concentrations by qT-RFLP analysis. The latter suggests an uptake of other substrates available for growth in the culture medium, e.g. amino acids. Reduced viability levels during exponential growth phase, as observed most prominently for *S. aureus*, may be caused by PI false positive stained cells. This can probably be attributed to the entry of PI due to short-term perforation of cell walls during cell division and cell wall synthesis [13,56]. Additionally, for *S. aureus*, aggregates of viable and dead cells might also increase total number of damaged bacteria due to clumping during growth [57].

Mixed culture cultivation impaired viability of *B. cepacia* and *S. aureus*, but did not affect viability of *P. aeruginosa*. For *B. cepacia*, viability in mixed cultures was reduced slightly compared to pure cultures from 12 h to 32 h. This may be caused by action of inhibitory pyocins produced by *P. aeruginosa*, as discussed above. For *S. aureus*, clear differences in the time course of viability in mixed and pure cultures were found. In mixed culture, viability decreased earlier and initially at a faster rate than in pure culture. This can most probably be explained by antagonistic interactions from Gram-negative bacteria towards *S. aureus*, most likely from *P. aeruginosa* due to dominance with regard to cell concentrations in mixed culture. Antistaphylococcal action can be transmitted by expressed virulence factors, e.g. pyocyanine or LasA protease, as discussed above. However, earlier reduction in viability of *S. aureus* in mixed culture may be additionally caused by other virulence factors, e.g. peptidoglycan hydrolases produced by *B. cepacia*[53]. Moreover, differences in dynamics of damaged and dead cells of *S. aureus* between pure and mixed culture clearly suggest the presence of various damaging processes in mixed culture, differing at least in time of action, which finally lead to a loss of membrane integrity. In general, the detection of damaged *S. aureus* cells might reflect a slower damaging process of membranes than for *B. cepacia* and *P. aeruginosa*, which is most probably due to differences in cell wall structures. Interestingly, for *B. cepacia* and *S. aureus* in mixed culture, a simultaneous increase of relative frequency of dead cells and a decrease of cell concentration was observed. These results suggest co-occurrence of cell dead (loss of membrane integrity) and lysis, which would further support simultaneous action of a set of various antagonistic interspecies effects during co-cultivation, differing in mode and time of action. Despite of this, synergistic interactions between the species cannot be excluded. For instance, Riedel et al. reported in their study of increased pathogenicity of *B. cepacia* induced by *P. aeruginosa* via quorum sensing signaling [58]. However, proteome data presented in a recently published report of Kluge et al. suggested no synergistic interspecies effects between the three species of interest in a similar mixed culture [46].

In the presented study, iron availability in the culture medium was not analyzed, although it might have a certain impact on growth and viability of single species in mixed culture. Iron limitation in medium in mixed culture will probably evoke interspecies effects for competition for remaining iron. Moreover, the expression of virulence factors might be regulated based on iron occurrence, as reported for *P. aeruginosa* for a large number of factors [59,60]. The impact of iron availability on growth and viability in the mixed culture remains to be clarified in further studies.

Overall, *P. aeruginosa* clearly dominated the mixed culture after 6 h of cultivation, with regard to the obtained cell concentrations. This emphasizes the predominance of *P. aeruginosa* over *B. cepacia* and *S. aureus* in the mixed culture under the chosen cultivation conditions, particularly due to very efficient substrate consumption of *P. aeruginosa*. Additionally, obtained growth and viability results suggest predominance of *P. aeruginosa* by induced antagonistic interspecies effects against *B. cepacia* and *S. aureus.* Interestingly, in patients with CF disease, *P. aeruginosa* is also often found to dominate late-term lung infections. This suggests that *P. aeruginosa* is dominant over *B. cepacia* and *S. aureus* in mixed community under a variety of growth conditions.

## Conclusion

A three-species mixed culture comprising *P. aeruginosa*, *B. cepacia* and *S. aureus* was characterized using a combination of analytical assays for monitoring species-specific cell concentrations and viabilities as well as determination of substrates and extracellular metabolites in the culture medium. The approach allowed for a comprehensive description of mixed culture dynamics of bacteria relevant to lung infections of CF patients, and enabled the identification of interspecies effects. In addition, the characterization of species-specific viability by flow cytometry provided insights into dynamics of cell damage in mixed cultures.

## Competing interests

The authors declare that they have no competing interests.

## Authors’ contributions

MR designed the study, did the laboratory work, analyzed the data and wrote the manuscript. MA participated in laboratory work and data analysis. UR supervised the study and helped to draft the manuscript. All authors read and approved the final manuscript.

## References

[B1] Hullar MAJ Kaplan LA Stahl DA Recurring seasonal dynamics of microbial communities in stream habitats Appl Environ Microbiol 2006 72 1 713 722 10.1128/AEM.72.1.713-722.2006 16391111 PMC1352240

[B2] Katsivela E Moore E Maroukli D Strömpl C Pieper D Kalogerakis N Bacterial community dynamics during in-situ bioremediation of petroleum waste sludge in landfarming sites Biodegradation 2005 16 2 169 180 10.1007/s10532-004-4883-y 15730027

[B3] Kleinsteuber S Schleinitz KM Breitfeld J Harms H Richnow HH Vogt C Molecular characterization of bacterial communities mineralizing benzene under sulfate-reducing conditions FEMS Microbiol Ecol 2008 66 1 143 157 10.1111/j.1574-6941.2008.00536.x 18637040

[B4] Liu WT Marsh TL Cheng H Forney LJ Characterization of microbial diversity by determining terminal restriction fragment length polymorphisms of genes encoding 16S rRNA Appl Environ Microbiol 1997 63 11 4516 4522 9361437 10.1128/aem.63.11.4516-4522.1997 PMC168770

[B5] Rogers GB Hart CA Mason JR Hughes M Walshaw MJ Bruce KD Bacterial diversity in cases of lung infection in cystic fibrosis patients: 16S ribosomal DNA (rDNA) length heterogeneity PCR and 16S rDNA terminal restriction fragment length polymorphism profiling J Clin Microbiol 2003 41 8 3548 3558 10.1128/JCM.41.8.3548-3558.2003 12904354 PMC179861

[B6] Thies FL König W König B Rapid characterization of the normal and disturbed vaginal microbiota by application of 16S rRNA gene terminal RFLP fingerprinting J Med Microbiol 2007 56 6 755 761 10.1099/jmm.0.46562-0 17510259

[B7] Trotha R Reichl U Thies FL Sperling D König W König B Adaption of a fragment analysis technique to an automated high-throughput multicapillary electrophoresis device for the precise qualitative and quantitative characterization of microbial communities Electrophoresis 2002 23 7–8 1070 1079 11981854 10.1002/1522-2683(200204)23:7/8<1070::AID-ELPS1070>3.0.CO;2-H

[B8] Schmidt JK König B Reichl U Characterization of a three bacteria mixed culture in a chemostat: evaluation and application of a quantitative terminal-restriction fragment length polymorphism (T-RFLP) analysis for absolute and species specific cell enumeration Biotechnol Bioeng 2007 96 4 738 756 10.1002/bit.21147 16937400

[B9] Schmidt JK Riedele C Regestein L Rausenberger J Reichl U A novel concept combining experimental and mathematical analysis for the identification of unknown interspecies effects in a mixed culture Biotechnol Bioeng 2011 108 8 1900 1911 10.1002/bit.23126 21391206

[B10] Riedele C Reichl U Interspecies effects in a ceftazidime-treated mixed culture of Pseudomonas aeruginosa, Burkholderia cepacia and Staphylococcus aureus: analysis at the single-species level J Antimicrob Chemother 2011 66 1 138 145 10.1093/jac/dkq394 21062793

[B11] Rogers GB Stressmann FA Koller G Daniels T Carroll MP Bruce KD Assessing the diagnostic importance of nonviable bacterial cells in respiratory infections Diagn Microbiol Infect Dis 2008 62 2 133 141 10.1016/j.diagmicrobio.2008.06.011 18692341

[B12] Nocker A Sossa-Fernandez P Burr MD Camper AK Use of propidium monoazide for live/dead distinction in microbial ecology Appl Environ Microbiol 2007 73 16 5111 5117 10.1128/AEM.02987-06 17586667 PMC1951001

[B13] Sträuber H Müller S Viability states of bacteria—specific mechanisms of selected probes Cytometry A 2010 77A 7 623 634 10.1002/cyto.a.20920 20583280

[B14] Fittipaldi M Nocker A Codony F Progress in understanding preferential detection of live cells using viability dyes in combination with DNA amplification J Microbiol Methods 2012 91 2 276 289 10.1016/j.mimet.2012.08.007 22940102

[B15] Hammes F Berney M Egli T Müller S, Bley T Cultivation-independent assessment of bacterial viability High Resolution Microbial Single Cell Analytics 2011 Heidelberg, Berlin: Springer 123 150 [Scheper T (Series Editor): *Advances in biochemical engineering/biotechnology*, volume 124.] 10.1007/10_2010_95 21069588

[B16] Gregori G Citterio S Ghiani A Labra M Sgorbati S Brown S Denis M Resolution of viable and membrane-compromised bacteria in freshwater and marine waters based on analytical flow cytometry and nucleic acid double staining Appl Environ Microbiol 2001 67 10 4662 4670 10.1128/AEM.67.10.4662-4670.2001 11571170 PMC93217

[B17] Alonso-Sáez L Gasol JM Lefort T Hofer J Sommaruga R Effect of natural sunlight on bacterial activity and differential sensitivity of natural bacterioplankton groups in northwestern mediterranean coastal waters Appl Environ Microbiol 2006 72 9 5806 5813 10.1128/AEM.00597-06 16957198 PMC1563624

[B18] Berney M Hammes F Bosshard F Weilenmann H-U Egli T Assessment and interpretation of bacterial viability by using the LIVE/DEAD BacLight Kit in combination with flow cytometry Appl Environ Microbiol 2007 73 10 3283 3290 10.1128/AEM.02750-06 17384309 PMC1907116

[B19] Bensch G Rüger M Wassermann M Weinholz S Reichl U Cordes C Flow cytometric viability assessment of lactic acid bacteria starter cultures produced by fluidized bed drying Appl Microbiol Biotechnol 2014 DOI 10.1007/s00253-014-5592-z 24584512 10.1007/s00253-014-5592-z

[B20] Falcioni T Papa S Gasol JM Evaluating the flow-cytometric nucleic acid double-staining protocol in realistic situations of planktonic bacterial death Appl Environ Microbiol 2008 74 6 1767 1779 10.1128/AEM.01668-07 18223113 PMC2268295

[B21] Foladori P Bruni L Tamburini S Ziglio G Direct quantification of bacterial biomass in influent, effluent and activated sludge of wastewater treatment plants by using flow cytometry Water Res 2010 44 13 3807 3818 10.1016/j.watres.2010.04.027 20537673

[B22] Johnson DR Czechowska K Chèvre N Van Der Meer JR Toxicity of triclosan, penconazole and metalaxyl on Caulobacter crescentus and a freshwater microbial community as assessed by flow cytometry Environ Microbiol 2009 11 7 1682 1691 10.1111/j.1462-2920.2009.01893.x 19239485

[B23] Ziglio G Andreottola G Barbesti S Boschetti G Bruni L Foladori P Villa R Assessment of activated sludge viability with flow cytometry Water Res 2002 36 2 460 468 10.1016/S0043-1354(01)00228-7 11827352

[B24] Shapiro HM Practical Flow Cytometry 2003 4 New York: Wiley-Liss 306 307

[B25] Zipper H Brunner H Bernhagen J Vitzthum F Investigations on DNA intercalation and surface binding by SYBR Green I, its structure determination and methodological implications Nucleic Acids Res 2004 32 12 e103 e103 10.1093/nar/gnh101 15249599 PMC484200

[B26] Rüger M Bensch G Tüngler R Reichl U A flow cytometric method for viability assessment of Staphylococcus aureus and Burkholderia cepacia in mixed culture Cytometry A 2012 81A 12 1055 1066 10.1002/cyto.a.22219 23081865

[B27] Müller S Nebe-von-Caron G Functional single-cell analyses: flow cytometry and cell sorting of microbial populations and communities FEMS Microbiol Rev 2010 34 4 554 587 20337722 10.1111/j.1574-6976.2010.00214.x

[B28] Riedele C Reichl U Time-kill studies with a ceftazidime-treated mixed culture consisting of Pseudomonas aeruginosa, Burkholderia cepacia and Staphylococcus aureus. Eng Life Sci 2012 12 2 188 197 10.1002/elsc.201100147

[B29] Holm C Jespersen L A flow-cytometric gram-staining technique for milk-associated bacteria Appl Environ Microbiol 2003 69 5 2857 2863 10.1128/AEM.69.5.2857-2863.2003 12732558 PMC154518

[B30] Gensberger ET Sessitsch A Kostić T Propidium monoazide–quantitative polymerase chain reaction for viable Escherichia coli and Pseudomonas aeruginosa detection from abundant background microflora Anal Biochem 2013 441 1 69 72 10.1016/j.ab.2013.05.033 23756735

[B31] Løvdal T Hovda MB Björkblom B Møller SG Propidium monoazide combined with real-time quantitative PCR underestimates heat-killed Listeria innocua J Microbiol Methods 2011 85 2 164 169 10.1016/j.mimet.2011.01.027 21324348

[B32] Pan Y Breidt F Enumeration of viable Listeria monocytogenes cells by real-time PCR with propidium monoazide and ethidium monoazide in the presence of dead cells Appl Environ Microbiol 2007 73 24 8028 8031 10.1128/AEM.01198-07 17933922 PMC2168130

[B33] Yáñez MA Nocker A Soria-Soria E Múrtula R Martínez L Catalán V Quantification of viable Legionella pneumophila cells using propidium monoazide combined with quantitative PCR J Microbiol Methods 2011 85 2 124 130 10.1016/j.mimet.2011.02.004 21329735

[B34] Nebe-von-Caron G Badley RA Viability assessment of bacteria in mixed populations using flow cytometry J Microsc 1995 179 55 66 10.1111/j.1365-2818.1995.tb03612.x

[B35] Barbesti S Citterio S Labra M Baroni MD Neri MG Sgorbati S Two and three-color fluorescence flow cytometric analysis of immunoidentified viable bacteria Cytometry 2000 40 3 214 218 10.1002/1097-0320(20000701)40:3<214::AID-CYTO6>3.0.CO;2-M 10878564

[B36] Van Dilla MA Langlois RG Pinkel D Yajko D Hadley WK Bacterial characterization by flow cytometry Science 1983 220 4597 620 622 10.1126/science.6188215 6188215

[B37] Debray H Decout D Strecker G Spik G Montreuil J Specificity of twelve lectins towards oligosaccharides and glycopeptides related to N-glycosylproteins Eur J Biochem 1981 117 1 41 51 7262089 10.1111/j.1432-1033.1981.tb06300.x

[B38] Sizemore RK Caldwell JJ Kendrick AS Alternate gram-staining technique using a fluorescent lectin Eur J Biochem 1990 56 7 2245 2247 10.1128/aem.56.7.2245-2247.1990 PMC184591 1697149

[B39] Forsgren A Sjöquist J “Protein a” from S. aureus: I. Pseudo-immune reaction with human γ-globulin J Immunol 1966 97 6 822 827 4163007

[B40] Inganäs M Johansson SGO Bennich HH Interaction of human polyclonal IgE and IgG from different species with protein a from Staphylococcus aureus: demonstration of protein-a-reactive sites located in the Fab2 fragment of human IgG Scand J Immunol 1980 12 1 23 31 10.1111/j.1365-3083.1980.tb00037.x 6158089

[B41] Ljungberg UK Jansson B Niss U Nilsson R Sandberg BEB Nilsson B The interaction between different domains of staphylococcal protein a and human polyclonal IgG, IgA, IgM and F (ab’)2: separation of affinity from specificity Mol Immunol 1993 30 14 1279 1285 10.1016/0161-5890(93)90044-C 8413328

[B42] Hughes EE Gilleland HE Jr Matthews-Greer JM Analysis by flow cytometry of surface-exposed epitopes of outer membrane protein F of Pseudomonas aeruginosa Can J Microbiol 1996 42 8 859 862 10.1139/m96-109 8776855

[B43] Nakayama K Takashima K Ishihara H Shinomiya T Kageyama M Kanaya S Ohnishi M Murata T Mori H Hayashi T The R-type pyocin of Pseudomonas aeruginosa is related to P2 phage, and the F-type is related to lambda phage Mol Microbiol 2000 38 2 213 231 10.1046/j.1365-2958.2000.02135.x 11069649

[B44] Waite RD Curtis MA Pseudomonas aeruginosa PAO1 pyocin production affects population dynamics within mixed-culture biofilms J Bacteriol 2009 191 4 1349 1354 10.1128/JB.01458-08 19060137 PMC2631993

[B45] Bakkal S Robinson SM Ordonez CL Waltz DA Riley MA Role of bacteriocins in mediating interactions of bacterial isolates taken from cystic fibrosis patients Microbiology 2010 156 7 2058 2067 10.1099/mic.0.036848-0 20378653 PMC3068677

[B46] Kluge S Hoffmann M Benndorf D Rapp E Reichl U Proteomic tracking and analysis of a bacterial mixed culture Proteomics 2012 12 12 1893 1901 10.1002/pmic.201100362 22623171

[B47] Machan ZA Taylor GW Pitt TL Cole PJ Wilson R 2-Heptyl-4-hydroxyquinoline N-oxide, an antistaphylococcal agent produced by Pseudomonas aeruginosa J Antimicrob Chemother 1992 30 5 615 623 10.1093/jac/30.5.615 1493979

[B48] Voggu L Schlag S Biswas R Rosenstein R Rausch C Götz F Microevolution of cytochrome bd oxidase in staphylococci and its implication in resistance to respiratory toxins released by pseudomonas J Bacteriol 2006 188 23 8079 8086 10.1128/JB.00858-06 17108291 PMC1698191

[B49] Kovárová-Kovar K Egli T Growth kinetics of suspended microbial cells: from single-substrate-controlled growth to mixed-substrate kinetics Microbiol Mol Biol Rev 1998 62 3 646 666 9729604 10.1128/mmbr.62.3.646-666.1998 PMC98929

[B50] Baron SS Rowe JJ Antibiotic action of pyocyanin Antimicrob Agents Chemother 1981 20 6 814 820 10.1128/AAC.20.6.814 6798928 PMC181804

[B51] Biswas L Biswas R Schlag M Bertram R Götz F Small-colony variant selection as a survival strategy for Staphylococcus aureus in the presence of Pseudomonas aeruginosa Appl Environ Microbiol 2009 75 21 6910 6912 10.1128/AEM.01211-09 19717621 PMC2772425

[B52] Kessler E Safrin M Olson JC Ohman DE Secreted LasA of Pseudomonas aeruginosa is a staphylolytic protease J Biol Chem 1993 268 10 7503 7508 8463280

[B53] Allan ND Kooi C Sokol PA Beveridge TJ Putative virulence factors are released in association with membrane vesicles from Burkholderia cepacia Can J Microbiol 2003 49 10 613 624 10.1139/w03-078 14663495

[B54] Lessie TG Phibbs PV Alternative pathways of carbohydrate utilization in pseudomonads Annu Rev Microbiol 1984 38 1 359 388 10.1146/annurev.mi.38.100184.002043 6388497

[B55] Roberts BK Midgley M Dawes EA The metabolism of 2-oxogluconate by Pseudomonas aeruginosa J Gen Microbiol 1973 78 2 319 329 10.1099/00221287-78-2-319 4202784

[B56] David F Berger A Hansch R Rohde M Franco-Lara E Single cell analysis applied to antibody fragment production with Bacillus megaterium: development of advanced physiology and bioprocess state estimation tools Microb Cell Fact 2011 10 1 23 10.1186/1475-2859-10-23 21496219 PMC3101136

[B57] Shapiro HM Multiparameter flow cytometry of bacteria: Implications for diagnostics and therapeutics Cytometry 2001 43 3 223 226 10.1002/1097-0320(20010301)43:3<223::AID-CYTO1054>3.0.CO;2-R 11170111

[B58] Riedel K Hentzer M Geisenberger O Huber B Steidle A Wu H Høiby N Givskov M Molin S Eberl L N-Acylhomoserine-lactone-mediated communication between Pseudomonas aeruginosa and Burkholderia cepacia in mixed biofilms Microbiology 2001 147 12 3249 3262 11739757 10.1099/00221287-147-12-3249

[B59] Kim E-J Wang W Deckwer W-D Zeng A-P Expression of the quorum-sensing regulatory protein LasR is strongly affected by iron and oxygen concentrations in cultures of Pseudomonas aeruginosa irrespective of cell density Microbiology 2005 151 4 1127 1138 10.1099/mic.0.27566-0 15817780

[B60] Ochsner UA Wilderman PJ Vasil AI Vasil ML GeneChip® expression analysis of the iron starvation response in Pseudomonas aeruginosa: identification of novel pyoverdine biosynthesis genes Mol Microbiol 2002 45 5 1277 1287 10.1046/j.1365-2958.2002.03084.x 12207696

